# Post-Critical Period Transcriptional and Physiological Adaptations of Primary Sensory Cortex after Sensory Loss

**DOI:** 10.1523/JNEUROSCI.1519-25.2025

**Published:** 2026-01-09

**Authors:** Laxmi Iyer, Kory Johnson, Sean Collier, Alan P. Koretsky, Emily Petrus

**Affiliations:** ^1^Henry M. Jackson Foundation for the Advancement of Military Medicine, Bethesda, Maryland 20817; ^2^Department of Anatomy, Physiology, and Genetics, Neuroscience Program, Uniformed Services University of the Health Sciences, Bethesda, Maryland 20814; ^3^Bioinformatics Core, Division of Intramural Research, National Institute of Neurological Disorders and Stroke, National Institutes of Health, Bethesda, Maryland 20892; ^4^Laboratory of Functional and Molecular Imaging, National Institute of Neurological Disorders and Stroke, National Institutes of Health, Bethesda, Maryland 20892

**Keywords:** denervation, sensory circuits, synaptic plasticity, thalamocortical plasticity, transcriptomics

## Abstract

Single cell transcriptomics supports both cell-specific characterization and the identification of responses to changes in neural activity. Unilateral whisker denervation in adult male and female mice activates post-critical period synaptic plasticity, but the transcriptional responses of neuronal subtypes remain unknown. Single nucleus RNA sequencing and multiplex fluorescence in situ hybridization identified previously unexplored plasticity mechanisms in layer 4 (L4) excitatory neurons in intact sensory cortex. We detected differentially expressed genes related to glutamate receptor signaling and synaptogenesis in thalamocortical (TC) recipient L4 sensory cortex neurons after whisker denervation. L4 excitatory neurons increase expression of glutamate receptors indicative of stabilized and potentiated TC synapses along the intact pathway. Immunohistochemistry and electrophysiology determined that intracortical connections to L4 neurons were specifically increased. Transcriptionally unique subtypes of L4 neurons responded uniformly to whisker denervation, likely responding to the global upregulation of activity in the intact sensory cortex after unilateral whisker denervation. These adaptations likely underlie the increased cortical activity responding to intact sensory inputs that are observed in rodents and humans after unilateral denervation injury.

## Significance Statement

The adult brain has a remarkable ability to adapt after changes in sensory experience or injury. Here we identified plasticity-related gene programs that were activated in layer 4 neurons after loss of sensory afferents. These changes occurred in similar ways despite the identified heterogeneity of these cell types. These findings help neuroscientists characterize how the adult brain adapts after sensory loss.

## Introduction

Sensory experience alters neuronal and synaptic function (Fox and Wong, 2005; Holtmaat and Svoboda, 2009), which often depends on transcriptional events in individual neurons within sensory circuitry. The advent of single cell transcriptomics has enabled neuroscientists to characterize transcriptionally distinct subpopulations of cells to complement the cell-type categorization that was previously achieved by morphology, function, and location (Scala et al., 2019). Recent studies indicate that transcriptionally unique neurons occupying a spatially similar region can play divergent roles in social behavior (Lischinsky et al., 2023; Walker and Frost, 2024), while others have demonstrated that morphology and spatial location play a larger role in sensory processing compared with transcriptional identity (Shainer et al., 2025). It is likely that a combination of these factors influence neural signaling to yield optimal brain function (Cembrowski et al., 2018), yet how transcriptional heterogeneity influences neuronal responses to altered sensory experience in the adult brain is only beginning to be appreciated.

The rodent whisker sensory system is a powerful model to study post-critical period plasticity in the adult brain. While neurons in superficial layers 2/3 remain highly plastic in adult rodents (Kole et al., 2017; 2018; Condylis et al., 2022; Butrus et al., 2025), thalamocortical (TC) connections to layer 4 (L4) neurons have traditionally been considered aplastic after the first postnatal week's “critical period”(Woolsey and Wann, 1976; Fox, 1992). However, recent studies have shown that changes in experience, driven by injury or sensory deprivation, can revive plasticity capacity in L4 neurons receiving TC input in multiple sensory systems (Montey and Quinlan, 2011; Yu et al., 2012; Petrus et al., 2014; Chung et al., 2017). In this study, we employed single nucleus RNA sequencing (snRNA-seq) to identify the molecular mechanisms that accompany post-critical period plasticity after unilateral whisker denervation via infraorbital nerve transection (ION-X).

SnRNA-seq identified differentially expressed genes (DEGs) in L4 excitatory neurons, which were confirmed with multiplex fluorescent in situ hybridization (mFISH). Functional consequences were identified with whole-cell electrophysiological recordings and protein detection with immunohistochemistry, providing multi-modal validation of molecular and functional changes. We determined that although snRNA-seq identified multiple subtypes of L4 excitatory neurons, functional adaptations after ION-X were largely uniform. After ION-X, L4 excitatory neurons receiving intact whisker sensory inputs exhibited increased glutamatergic receptor function and intracortical synaptogenesis. These combined adaptations likely stabilize the TC synapses and enhance responses to compensate for the contralateral sensory loss. Further, the finding that transcriptionally unique excitatory L4 neuronal subtypes exhibit similar adaptations to sensory deprivation highlights the important influence of neuronal location and global circuit function rather than transcriptional identity alone.

## Materials and Methods

### Animals

All procedures were approved by the National Institutes of Health Animal Care and Use committee (ACUC) under protocol 1160 and the Uniformed Services of the Health Sciences Institutional Animal Care and Use Committee (IACUC) under protocol APG-22-081. Facilities in both universities are accredited by the association for assessment and accreditation of laboratory animal care (AAALAC). C57Bl/6j mice (Strain#000664, RRID: IMSR_JAX:000664) were obtained from Jackson Laboratories and arrived on site at 4–6 weeks of age. SnRNA-seq experiments included only male mice but subsequent multiplex fluorescent in situ hybridization (mFISH), immunohistochemistry, and electrophysiological experiments were male/female balanced. No statistically significant differences were identified between sexes. Littermates were housed 2–4 per cage with food and water *ad libitum* on a 12 h light/dark cycle. Breeder mice were purchased from Jackson Laboratories including B6;C3-Tg(Scnn1a-cre)3Aibs/J and B6.Cg-*Gt(ROSA)26Sor^tm14(CAG-tdTomato)Hze^*/J and bred to produce homozygous offspring. Their genotypes were validated by PCR genotyping before weaning.

### Surgery

All surgeries were performed using aseptic techniques. After each surgery, animals received Meloxicam slow release to combat inflammation. Animals remained on a heated surface and returned to the animal facility after they were fully ambulatory, typically within 20–60 min.

#### Whisker denervation surgery (ION-X)

After 1 week of habituation to in-house animal facilities, 4–6-week-old mice were anesthetized with an intraperitoneal (i.p.) injection of a ketamine/xylazine/saline cocktail (80 mg/kg ketamine, 10 mg/kg xylazine). Once mice were unresponsive to paw pinch, the right side of the face was shaved, and an incision caudal to the whiskerpad was made to visualize the infraorbital nerve (ION) bundle. For sham mice, the procedure was concluded at this step, with the incision closed with a single suture and a drop of tissue glue (Tissuemend II, Veterinary Product Laboratories). In ION-X mice, the ION bundle was cut with scissors, and the incision was closed as described above. Animals then received an intraperitoneal injection of Antisedan (1 mg/kg; Atipamezole, Zoetis) to reverse the anesthesia.

#### Stereotaxic surgery

At 3–4 weeks of age, mice were anesthetized with 1–3% isoflurane mixed with O_2_ and unilaterally injected (left side) with 500 nl of a virus encoding for channel rhodopsin (ChR2): AAV2/9.hSynapsin.hChR2(H134R)-EYFP.WPRE.hGH (RRID: Addgene_26973, titer 2.1 × 10^13^). Animals received the viral injection into the sensory thalamus: ventral posteromedial nucleus (VPM). Coordinates were ML 1.6, AP 2.0, depth 3.5. The virus was delivered with a 2.5 µl Hamilton syringe with a 30 G beveled needle. Injection duration was 10 min with the needle left in place for 10 additional minutes to ensure adequate perfusion into the tissue. The incision was closed with tissue staples. These mice were used for electrophysiology recordings.

### Single nucleus RNA sequencing

The protocol was followed according to Sathyamurthy et al. (2018).

#### Sample collection and processing

Twelve days after sham/ION-X surgery, male mice were anesthetized with 5% isoflurane until the absence of a righting reflex was observed. Mice were then decapitated and the intact (contralateral to the intact whiskers) S1BC was rapidly dissected into ice-cold dissection buffer (80 mM NaCl, 3.5 mM KCl, 1.25 mM H2PO_4_, 25 mM NaHCO_3_, 4.5 mM MgSO_4_, 0.5 mM CaCl_2_, 10 mM glucose, and 90 mM sucrose), which was bubbled continuously with a 95% O_2_/5% CO_2_ gas mixture. Two S1BCs (one per mouse) were collected per sample, with an average collection time of 2–4 min. Each sample was dounced using five strokes with Pestle A, followed by five strokes with Pestle B (Kimble: Kontes Dounce Tissue Grinder) in sucrose buffer containing the following: 0.32 M sucrose, 10 mM HEPES, pH 8.0, 5 mM CaCl_2_, 3 mM Mg-acetate, 0.1 mM EDTA, 1 mM dithiothreitol, 0.1% Triton X-100. The lysate was diluted with 3 ml of sucrose buffer and centrifuged at 3,200 × *g* for 10 min. The supernatant was removed and 3 ml sucrose buffer was added, followed by a 2 min incubation. The pellet and sucrose were transferred to an Oak Ridge centrifuge tube, where the pellet was homogenized using an Ultra-Turrax (IKA Instruments) for 1 min at setting 1. Then, 12 ml of density buffer (1 M sucrose, 10 mM HEPES, 3 mM Mg-acetate, 1 mM dithiothreitol) was added below the layer of nuclei. The tube was centrifuged again at 3,200 × *g* for 20 min. The supernatant was poured off, and the nuclei were collected from the walls of the tube with 1 ml PBS plus 0.02% bovine serum albumin (BSA). This solution was spun once more at 3,200 × *g* for 10 min. Nuclei were resuspended in PBS with 0.02% BSA. Samples were placed on ice and transferred to the National Human Genome Research Institute single cell genomics core facility for processing on 10× and Illumina platforms. 8 samples (4 sham, 4 ION-X, 2 mice per sample) were sequenced on three separate sample dates. The kits used are as follows: 10× Chromium Next Gen Chip G Single Cell Kit, 16rxns; Chromium Next Gem Automated Single Cell 3’ Library & Gel Bead Kit v3.1, 4rxns; Illumina Next-Seq 500/550 High Output Kit v2.5 (150 cycles). High output run with 400 M reads. The target input cell number was 10,000 nuclei per sample, with four samples per 400 M read experiment. The sequencing saturation was between 17.7 and 19.3%, there were ∼96% valid barcodes read and ∼108 million reads per sample; these are expected values for single nucleus RNA sequencing. The 10× Cell Ranger workflow (v7.0.0) was used to align sequences to the reference mouse genome. Single-nuclei sequencing is preferable over single-cell for this experiment as we are looking for plasticity-induced transcriptional changes, and this technique has less tissue handling and dissociation-related effects, leading to a more comprehensive picture of cell type proportions.

#### snRNA-seq data analysis

All snRNA-seq analysis was performed using R (version 4.4.1) in conjunction with functions supported in Seurat (version 5.1.0) unless otherwise specified. Filtered “h5” output files from Cell Ranger (Sample Collection and sequencing) were imported into R by sample using the “Read10X_h5” function. The “CreateSeuratObject” function was then applied to these files to produce one Seurat object per sample containing the total number of counts per nucleus (nCount_RNA) and the total number of detected genes per nucleus (nFeature_RNA). Mitochondrial gene content (percent.mt) was calculated using the “PercentageFeatureSet” function (pattern = “^mt-”). Individual Seurat objects were then collapsed into one using the “merge” function. After applying the above, the data were scrutinized by UMAP plot and found to not have spatial bias by sample or by year. We did not detect any gross bias per year or sample, arguing and supporting the fact that SCT integration performed well. The “VlnPlot” function was then applied to this collapsed object to visually compare the percent.mt, nCount_RNA, and nFeature_RNA values across samples (Fig. S1*A*). Nuclei were retained for downstream analysis if they passed quality control filters: percent.mt < 5, 675 < nCount_RNA <15,000, and 500 <nFeature_RNA < 5,000. Post filtering, the collapsed object was split back into individual objects by sample using the “SplitObject” function.

Each dataset of split-out object was then normalized using the “SCTransform” function (vars.to.regress = “percent.mt”, method = “glmGamPoi”). Integration of this now normalized data across objects into a single object was accomplished using the “SelectIntegrationFeatures” function (nfeatures = 3,000) and the “PrepSCTIntegration” function followed by use of the “FindIntegrationAnchors” and “IntegrateData” functions (normalization.method = “SCT”). Post integration, the “RunPCA” and “ElbowPlot” functions were applied to generate and explore the proportion of variance by each principal component (PC) up to 200 components (npcs = 200). Based on this analysis, top 100 PCs were selected for clustering. The “RunUMAP” function was used in conjunction with the “FindNeighbors” function (dims = 1:100), and the clustering was performed using “FindClusters” function over a range of resolutions (0.4:1.5). Clustering results were then inspected at each resolution using the “DimPlot” function (group.by = “UMAP”) as well as across resolutions using the “clustree” function. From this inspection, 0.80 was selected as the optimal resolution.

To remove doublets, “computeDoubletDensity” (https://plger.github.io/scDblFinder/articles/computeDoubletDensity.html) and “findDoubletClusters” (https://plger.github.io/scDblFinder/articles/findDoubletClusters.html) functions were used. A *z*-score was also calculated for each nucleus using its UMAP1 value in conjunction with the mean (trim = 0.20) and SD calculated from all UMAP1 values for nuclei that fall in the same cluster. Nuclei with *z*-score >2 were then filter removed as cluster-level outliers and the same procedure was repeated using UMAP2 values. These steps helped eliminate low-confidence and mis-clustered nuclei; no clusters were removed using this strategy. Eight samples were obtained on three separate dates (batches). The target number of nuclei was 10,000 nuclei per sample, and the distribution was as follows. Batch 1: Sham 10,978; ION-X 10,508. Batch 2: Sham1 11,853; Sham2 14,695; ION-X1 9,859; ION-X2 11,623. Batch 3: Sham 9,124; ION-X 10,017. The sequencing saturation was between 17.7 and 19.3%, there were ∼96% valid barcodes read, and ∼108 million reads per sample.

Surviving nuclei per cluster were then summarized by sample via stacked bar plot using the “ggplot” and “geom_bar” functions (position = “stack”). For cluster annotation, the “RunAzimuth” function was used (reference = “mousecortexref”). Canonical marker genes for each cluster was identified using the “FindConservedMarkers” function and was used to validate and refine the Azimuth assigned cell type. The “FeaturePlot” and “VlnPlot” (stack  =  TRUE) functions were used to visualize the marker gene expression. To rank prioritize clusters on differences occurring between sample conditions (i.e., ION-X vs Sham), the “augur” function (https://github.com/neurorestore/Augur) was used. To test for which genes are different between sample types within each cluster, the “FindMarkers” function was used (ident.1 = “ION”, ident.2 = “Sham”, logfc.threshold = 0). Results returned from this function included “avg_log2FC” and “p_val_adj” values which were used to rank sort and select candidate differential expressed genes. Genes that had a log2FC value of >0.1, adjusted *p* value of <0.05, and of physiological interest were selected for further analysis using multiplex fluorescent in situ hybridization, immunohistochemistry, and electrophysiology. We used a loose log2FC cutoff to have sufficient genes to run GO analysis and to identify genes that were testable with functional (electrophysiological) assays.

The above steps post integration were repeated two separate times on two cluster subsets:

1. Excitatory neurons with positive expression of *Rbfox3*, *Map2*, or *Slc17a7* were reclustered using 50 PCs and a resolution of 0.04.
2. L4 excitatory neurons with positive expression of *Map2*, *Slc17a7*, and *Rorb* were reclustered using 100 PCs and a resolution of 0.80.

Differentially expressed genes from L4 neurons with an adjusted *p* value of 0.05 or less were used to conduct Gene Ontology enrichment analysis using the clusterProfiler (4.62) package for R (3.16.0). Only L4 clusters were analyzed for the subsequent experiments, not L4/5. The mouse gene database—org.Mm.eg.db—was used as a reference to identify overrepresented biological processes in our gene list. The top 10–11 entries for each category are listed by *q* value and colored according to the number of DEGs included. The background genes used for GO analysis consist of all the genes collected for the cell type of interest. The number of genes for each group are as follows: L2/3: 12,294; L4: 11,897; L5: 12,897; L6: 11,951.

### Multiplex florescent in situ hybridization (mFISH)

The protocol was followed according to the manual for HiPlex12 Reagent RNAScope Assay (Advanced Cell Diagnostics, catalog #324419).

#### Sample preparation and hybridization

Twelve days after sham/ION-X surgery, C57BL6/J mice were anesthetized using isoflurane and transcardially perfused with ice-cold 1× PBS, followed by 4% paraformaldehyde (PFA). Whole brains were post-fixed in 4% PFA for 24 h at 4°C and then sequentially incubated in 10%, 20%, and 30% sucrose in PBS for 24 h each. The brains were then embedded in OCT, frozen, and stored at −80°C. Frozen S1BC sections of 12 µm were cut using cryostat (CM1950 Leica Biosystems) and collected onto SuperFrost Plus Slides. To prevent RNA degradation, the slides were immediately frozen at −20°C.

To validate the results from the snRNA-seq experiments and to visualize multiple gene candidates in one slice, we performed HiPlex12 RNAScope with combination of 6–12 different probes according to the protocol. The list of oligonucleotides used as target probes is in the Dataset S4–S2. The sections were briefly rinsed with PBS, baked at 60°C for 30 min, post-fixed with 4% PFA, and dehydrated through graded ethanol concentrations. The slides were then immersed in a target retrieval buffer in a steamer for 5 min and dried overnight circled with a hydrophobic barrier. The slides were treated with Protease III reagent and then subsequently with a pooled set of 6–12 HiPlex probes for 2 h at 40°C. Probes with different tails attached (T1–T12) allowed for specific and accurate detection of the desired gene. Sections were then treated with three signal amplifying solutions (Amp 1–3) for 30 min each, followed by 3% FFPE reagent to reduce autofluorescence. The last step was the addition of fluorophore for each of the corresponding channels—488, 550, and 650 nm—which allowed for the detection of three mRNA targets (T1–T3) in each round. DAPI was used as a counterstain and tiled images were captured on Zeiss AxioScan.Z1 microscope with a 40× objective.

After each round, the probes were cleaved using a 10% cleaving solution, and the next set of florescent detection reagents (T3–T6) was applied. After last round, the probes were cleaved again and a blank image was captured without any probes for background subtraction. All the rounds of images for each section were aligned and registered to HiPlex Image Registration Software v2.1.0 (ACDBio) and a final composite image with 6–12 probes was generated. Each probe was stained in 5–6 mice per group and two nonadjacent sections per mouse were analyzed.

#### Image analysis and quantification

Quantification of mRNA expression from the composite image obtained from the ACD HiPlex Registration software was performed utilizing QuPath (v0.4.3; Bankhead et al., 2017) bioimage software. For each section, three nonoverlapping rectangular ROIs (0.8 × 0.7 in) spanning intact S1BC were manually defined and used for quantification. Counts from the three ROIs were averaged for each section. For each ROI, first the nuclei were identified using the DAPI channel via QuPath's “positive cell detection” tool. Thresholds for DAPI signal intensity were manually set to optimize accurate detection of individual nuclei. The “subcellular detection” tool was then utilized to detect and quantify mRNA puncta in each channel based on florescent intensity. Detection thresholds for each gene were independently optimized based on signal-to-noise ratio. A nucleus was considered positive for a gene if it contained one or more puncta for that gene within the nuclear boundary (Erben and Buonanno, 2019). QuPath provided a table with cell-by-cell measurements of all the 12 targeted probes in each nucleus.

To specifically identify L4 excitatory neurons, we filtered nuclei based on coexpression of *Map2*, *Slc17a7*, and *Rorb*. The expression of these three markers was consistent across sham and ION-X samples, ensuring comparable selection of L4 neurons across groups. Expression of other gene targets was quantified only within the Map2+/Slc17a7+/Rorb+ nuclei, i.e., the L4 nuclei, using two complementary metrics:

1. Percentage of positive nuclei—This metric reflects the proportion of L4 nuclei that express a given target gene, calculated as the number of gene-positive nuclei out of the total number of nuclei within each ROI (typically ∼400 nuclei per ROI). This provides a population-level view of gene expression, indicating how widely a gene is expressed across L4 neurons in each condition.
2. Average puncta per nucleus—This measure captures the average number of mRNA puncta per individual L4 nucleus that is positive for the gene. It highlights differences in gene expression levels among positive cells, offering insight into the cell-to-cell variability in transcriptional activity. Together, these metrics differentiate between changes in the prevalence of gene expression (how many nuclei express it) and proportion of expression (how much of the transcript is present in each expressing cell), allowing for two types of gene regulation analysis between sham and ION-X conditions (Fig. S2*A–C*).

Each data point represents the average of two sections per mouse. Group comparisons between ION-X and sham were analyzed using unpaired two-tailed *t* tests, and results are reported as mean ± standard error of the mean (SEM). Statistical significance was considered at *p* < 0.05.

### Electrophysiological recordings

#### Acute slice preparation

Two weeks after ION-X or sham surgeries acute slices were made for whole-cell electrophysiological recordings. Mice were anesthetized using isoflurane vapors (5% mixed with O_2_) until the absence of the corneal reflex was observed. The brain was quickly dissected and immersed in ice-cold dissection buffer (80 mM NaCl, 3.5 mM KCl, 1.25 mM H_2_PO_4_, 25 mM NaHCO_3_, 4.5 mM MgSO_4_, 0.5 mM CaCl_2_, 10 mM glucose, and 90 mM sucrose), which was bubbled continuously with a 95% O_2_/5% CO_2_ gas mixture. Brain blocks containing primary somatosensory cortex were dissected and coronally sectioned into 300-µm-thick slices using a Leica VT1000S Vibratome (Leica Biosystems). Slices were incubated for 60 min at room temperature before recordings began.

#### Recordings

Slices were transferred to a submersion-style recording chamber mounted on a fixed stage (Sutter Instrument) with an upright Nikon Eclipse FN1 microscope (Nikon Instruments) and illuminated with oblique infrared (IR) illumination. Recordings were performed in artificial CSF (ACSF; 130 mM NaCl, 3.5 mM KCl, 1.25 mM NaH_2_PO_4_·H_2_O, 24 mM NaHCO_3_, 10 mM glucose, 2.5 mM CaCl_2_, and 1.5 mM MgCl_2_) bubbled with 95% O_2_/5% CO_2_ at 30°C. Then, 0.005 mM bicuculline and 0.1 mM APV were added to ACSF to isolate AMPA receptor-mediated excitatory events. The ACSF was perfused at a rate of 2 ml/min. Voltag-clamp experiments used Cs-gluconate internal solution, containing the following: 130 mM Cs-gluconate, 8 mM KCl, 1 mM EGTA, 10 mM HEPES, 4 mM ATP, 5 mM QX-314; pH 7.3, 285–295 mOsm. Neurons in L4 were targeted for patch-clamp recordings with an access resistance higher than 25 MΩ and input resistance lower than 100 MΩ were discarded. Cells which had more than a 20% fluctuation in these values for recordings were excluded from analysis. A maximum of three cells per animal, one cell per slice, were recorded for each dataset.

#### Rectification index

Then, 0.1 mM spermine (Tocris) was added to the internal solution. Monosynaptic EPSCs were evoked by 455 nm LED illumination of ChR2-expressing TC terminals. Light intensity was adjusted so events were ∼100 pA at −70 mV. EPSCs were recorded in pseudorandom order at holding potentials including the following: −70, −60, −20, 0, +20, and +40 mV at 0.1 Hz with 6–10 events per holding potential. The rectification index was calculated as EPSC amplitudes at +40 mV/−60 mV. Six sham and 6 ION-X mice were used to obtain 12 and 13 cells, respectively.

#### Philanthotoxin sensitivity

Monosynaptic EPSCs were evoked by 455 nm LED illumination of ChR2-expressing TC terminals. Light intensity was adjusted so events were ∼100 pA. A 5 min baseline at 0.1 Hz was recorded before the addition of 10 µM philanthotoxin 433 (PhTX433; Aobious). EPSCs were recorded at 0.1 Hz for up to 30 min after PhTX addition. The percent reduction was calculated as amplitude of post-PhTX EPSC/pre-PhTX EPSC. Five sham and 6 ION-X mice were used to obtain 9 and 9 cells, respectively.

#### Miniature excitatory post synaptic currents (mEPSCs)

Neurons in L4 were targeted for voltage-clamp recording and mEPSCs were recorded for 3–5 min. ACSF contained 1 µM tetrodotoxin (TTX), 20 µM bicuculline, and 100 µM dl-2-amino-5-phosphonopentanoic acid (dl-APV). A total of 200 consecutive mEPSCs were selected for analysis using MiniAnalysis software (BlueCell). Four sham and 4 ION-X mice were used to obtain 11 and 10 cells, respectively.

#### Analysis of electrophysiology data

An axon patch-clamp amplifier 700B (Molecular Devices) was used for patch-clamp recordings. Data were acquired through pClamp11 and analyzed with Clampfit 11.2 software (Molecular Devices). sEPSCs were analyzed using MiniAnalysis software by synaptosoft/BlueCell. Statistics were performed using unpaired Student's *t* test between sham versus ION-X. Values are depicted as mean ± SEM.

### Immunohistochemistry synapse imaging and quantification

#### Immunohistochemistry (IHC)

Scnn1a-cre3 mice were bred with ROSA-TdTomato mice to produce homozygous offspring which was validated by PCR genotyping at birth. This combination selectively labels excitatory cortical neurons in layer 4 and were used for this experiment. Fourteen days after sham or ION-X surgeries, mice were anesthetized and perfused transcardially with ice-cold PBS, followed by 4% PFA. Brains were post-fixed overnight in 4% PFA at 4°C, then 15%, and 30% sucrose in PBS and cryoprotected in OCT at −80°C. Coronal sections of 25 µm thickness containing S1BC were collected using a Leica Biosystems CM1950 cryostat.

Free-floating sections (three per animal) were washed three times for 10 min in 0.2% Triton X-100 in TBS (TBST) and then blocked in 10% normal goat serum (NGS) in PBST for 1 h at room temperature. Sections were incubated for 48 h at 4°C in primary antibodies diluted in 5% NGST: guinea pig anti-VGluT1 (1:2,000, AB5905, MilliporeSigma), guinea pig anti-VGluT2 (1:2,000, AB2251-I, MilliporeSigma), and rabbit anti-PSD95 (1:300, 51–6900, Invitrogen). After washing three times for 10 min in PBST, sections were incubated in 5% NGST with secondary antibodies for 2 h at room temperature, protected from light: goat anti-guinea pig Alexa 647 (1:200, A21450, Invitrogen) and goat anti-rabbit Alexa 488 (1:200, A21450, Invitrogen). Sections were mounted with Prolong Gold antifade medium and sealed with nail polish. All images were acquired within 48 h of mounting. High-resolution *z*-stacks of S1BC L4 were acquired using a Zeiss LSM 980 confocal microscope with a 63× oil-immersion objective (NA 1.4). For each section, 2–3 image stacks were acquired from L4 using tdTomato signal as a guide for cortical depth. *Z*-stacks consisted of 30 optical sections captured at a step size of 0.17 µm (total depth ≈ 5 µm), with a resolution of 1,024 × 1,024 pixels and 1.64× digital zoom. Imaging parameters were kept constant across all conditions and genotypes.

#### Synapse quantification

Synaptic puncta quantification was performed using SynBot, an open-source FIJI-based analysis pipeline designed for automated synapse detection and classification (Savage et al., 2024). Maximum projections of six consecutive optical sections (corresponding with 1 µm total depth) were generated from the original *z* stack. Nonoverlapping ROIs from three sections per mice were analyzed. The analysis was performed as per SynBot protocol (https://www.protocols.io/view/synbot-protocols-3byl4qewjvo5/v2). After installing the plugin, open the SynBot ijm plugin file in FIJI. Once the macro begins running, we chose two-channel localization along with the Pixel-overlap analysis type for Vglut1-PSD95 and Vglut2-PSD95 images. A summary excel file along with the overlays for synapses and puncta counted in each image is obtained. Average puncta count and colocalized synapse densities were compared between sham and ION-X groups using unpaired two-tailed *t* tests. Data was visualized as mean ± SEM.

### Statistical analyses

All the statistical analyses were performed in GraphPad Prism (10.5.0) or Microsoft Excel. Unpaired *t* tests were used to compare sham versus ION-X.

## Results

### Transcriptional profile of intact mouse primary somatosensory barrel cortex (S1BC)

**Figure 1.**
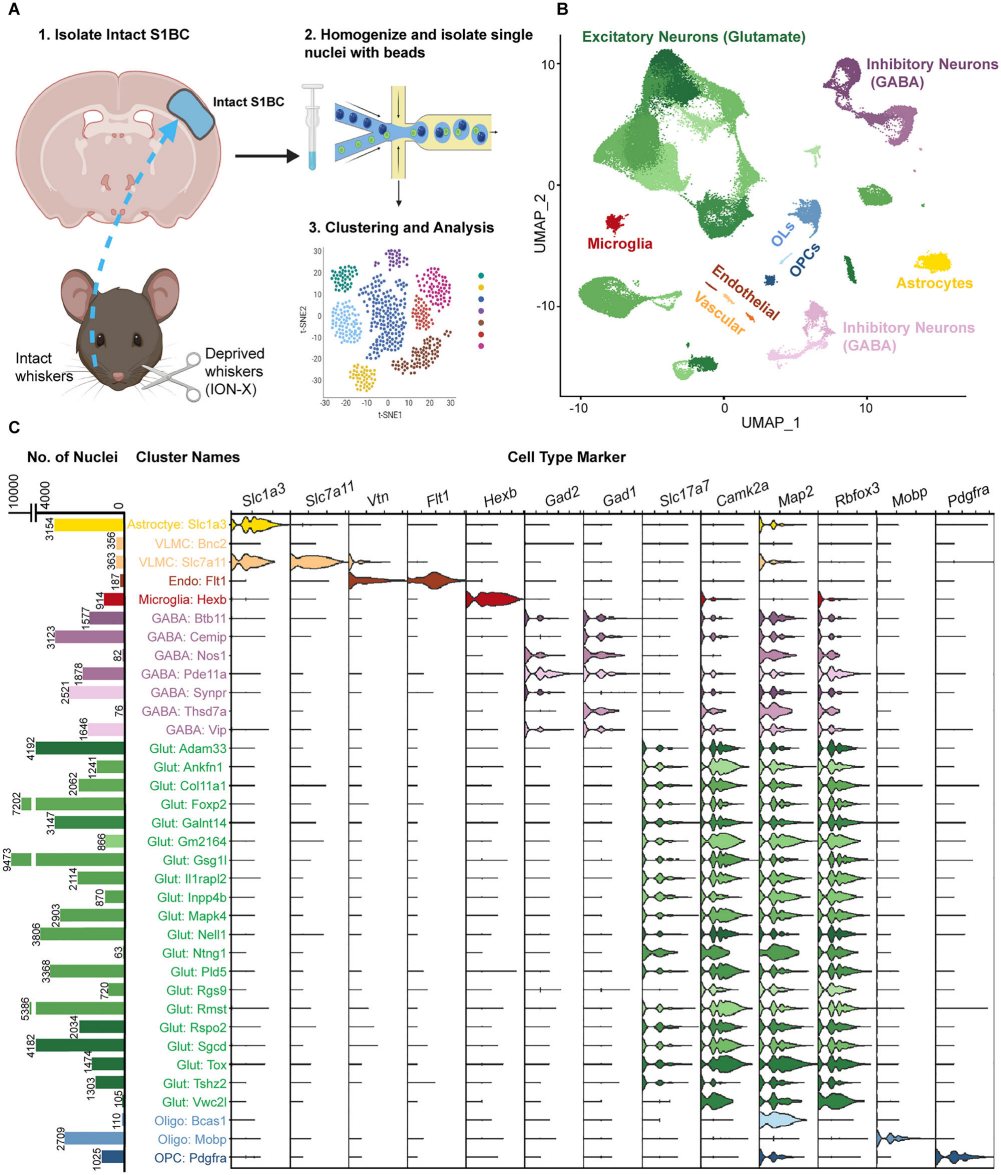
Transcriptional profile of intact mouse primary somatosensory barrel cortex (S1BC). ***A***, Schematic of snRNA-seq workflow (*n* = 8 C57BL6/J male mice/group), samples collected 12 d after sham or ION-X. ***B***, Uniform manifold approximation and projection (UMAP) of 83,390 nuclei grouped into 35 clusters, annotated by cell type. Oligodendrocytes (OLs) and oligodendrocyte progenitor cells (OPCs). ***C***, Left, Bar graphs representing numbers of nuclei per cluster. Right, Violin plots showing expression levels of canonical marker genes used to define major cell types. See also Figures S1; Dataset S1.

We collected intact (contralateral to- and receiving inputs from- the intact whisker set) S1BC brain tissue 12 d after sham or ION-X surgery in male mice (Fig. 1*A*). This timepoint was chosen because gene expression is predicted to precede the maximal potentiation of the intact S1BC’s response to injury (Chung et al., 2017). SnRNA-seq was performed, and the data were filtered to remove nuclei with low-quality reads, doublets, and >5% mitochondrial genes (Fig. S1*A*; Materials and Methods). A total of 83,390 nuclei were retained and grouped into 35 transcriptionally distinct clusters. Cell-type annotations were generated using expression of canonical marker genes for major cell types (Fig. 1*B*) and confirmed with automated alignment to the Allen Brain Atlas (Yao et al., 2021; Fig. S1*B*). These cell types and markers include the following: astrocytes (*Slc1a3*), excitatory neurons (*Slc17a7*, *Camk2a*, *Rbfox3*, *Map2*), inhibitory neurons (*Gad1*, *Gad2*, *Rbfox3*, *Map2*), microglia (*Hexb*), endothelial and vascular cells (*Flt1*, *Vtn*, *Slc7a11*), oligodendrocytes (*Mobp*), and oligodendrocyte progenitor cells (*Pdgfra*; Fig. 1*C*). The cluster distribution of nuclei was consistent between sham and ION-X samples, indicating all cell types were represented equally after ION-X (Fig. S1*A*, Dataset S1-1).

### Transcriptional characterization of excitatory layer 4 (L4) neurons receiving intact whisker inputs

**Figure 2.**
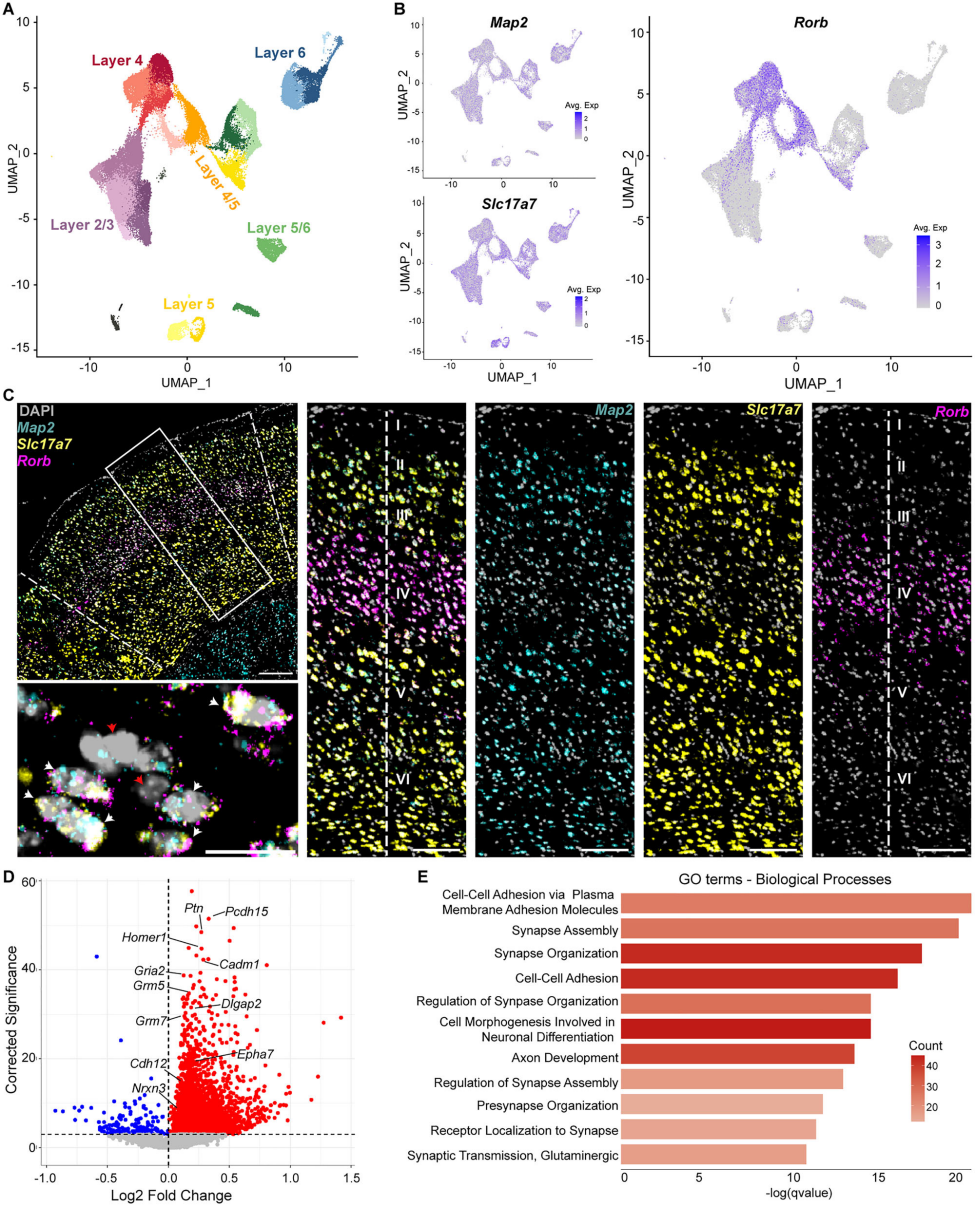
Transcriptional characterization of excitatory layer 4 (L4) neurons receiving intact whisker inputs. ***A***, UMAP of 76,232 reclustered excitatory neuronal nuclei annotated by cortical layer identity. ***B***, Feature plots for neuronal marker *Map2*, excitatory cell marker *Slc17a7* and Layer 4 marker *Rorb* expression used to define L4 excitatory neurons. ***C***, mFISH validation of L4 neurons in S1BC showing coexpression of *Map2*, *Slc17a7*, and *Rorb*, counter-stained with DAPI. Left, Composite image of S1BC with boxed area expanded at right to show cortical layers in split channels. Bottom left, Image showing L4 excitatory nuclei positive (white arrows) and negative (red arrows) for all three markers. Scale bars: 200 µm (top left), 10 µm (bottom left), 100 µm (right panels). ***D***, Volcano plot with differentially expressed genes (DEGs) in L4 excitatory neurons comparing sham and ION-X mice. ***E***, Gene Ontology (GO) enrichment analysis of upregulated biological processes, ranked by *q* value (adjusted *p* value), colored by number of DEGs. See also Dataset S3.

Excitatory neurons (76,232 nuclei) expressing *Rbfox3*, *Map2*, and *Slc17a7* (encoding for NeuN, Map2, and Vglut1 proteins, respectively) were identified (Fig. S1*C*), subset out, reclustered, and classified into 26 clusters. These clusters were manually annotated and assigned to cortical layers (Fig. 2*A*, Fig. S1E, Dataset S1–S3). Annotations were validated with atlas-aligned auto-annotation (Fig. S1*D*, Dataset S1–S3; Yao et al., 2021). L4 excitatory neurons were defined by coexpression of pan-neuronal marker *Map2*, excitatory cell marker *Slc17a7*, and L4-specific marker *Rorb* (Fig. 2*B*), which was confirmed with mFISH in S1BC (Fig. 2*C*). *Rorb* is a transcription factor that is required for the formation of barrels in L4 of rodent S1BC (Clark et al., 2020) and is conserved in sensory cortex of multiple species (Chen et al., 2023; Qian et al., 2025). Differential gene analysis revealed that 403 genes were significantly upregulated, and two genes were downregulated (adjusted *p* < 0.05) in L4 excitatory neurons in ION-X compared with sham mice (Fig. 2*D*, Dataset S3–S5). Gene Ontology (GO; Thomas et al., 2022) enrichment analysis detected upregulated pathways associated with synapse assembly and organization, cell–cell adhesion, and glutamate receptor signaling (Fig. 2*E*, Dataset S3–S6). These data suggest molecular and circuit remodeling in the intact whisker pathway and validate L4 excitatory neuron identification across both sequencing and mFISH platforms. Other clusters of excitatory neuron nuclei were inspected for differentially expressed genes (DEGs). Nuclei in layer 2/3 exhibited 1,081 upregulated and 8 downregulated genes, contributing to more than 20 GO pathways (Dataset S2-1). In contrast, layer 5 neurons expressed 18 upregulated and 0 downregulated genes, with no significant GO pathways identified (Dataset S2-2). Layer 6 neurons expressed 31 upregulated and 129 downregulated genes, with one GO pathway identified (Dataset S2-3). We excluded mixed clusters such as those with markers for L4/5 or 5/6. Layer 2/3 cortical neurons are known to remain highly dynamic throughout the lifespan (Jiang et al., 2007; Wen and Barth, 2011), so although they had the most numerous DEGs, we elected to focus on L4 and the thalamocortical (TC) pathway. Historically, this connection was considered aplastic after the critical period (Fox, 1992; Barth and Malenka, 2001); however, the loss of sensory inputs can recruit latent plasticity mechanisms in the adult brain (Montey and Quinlan, 2011; Petrus et al., 2014; Chung et al., 2017; Jie et al., 2025). Here we hypothesized that there may be additional post-critical period plasticity mechanisms after sensory loss that snRNA-seq would be able to detect.

### Altered glutamate receptor signaling in the intact sensory pathway after ION-X

**Figure 3.**
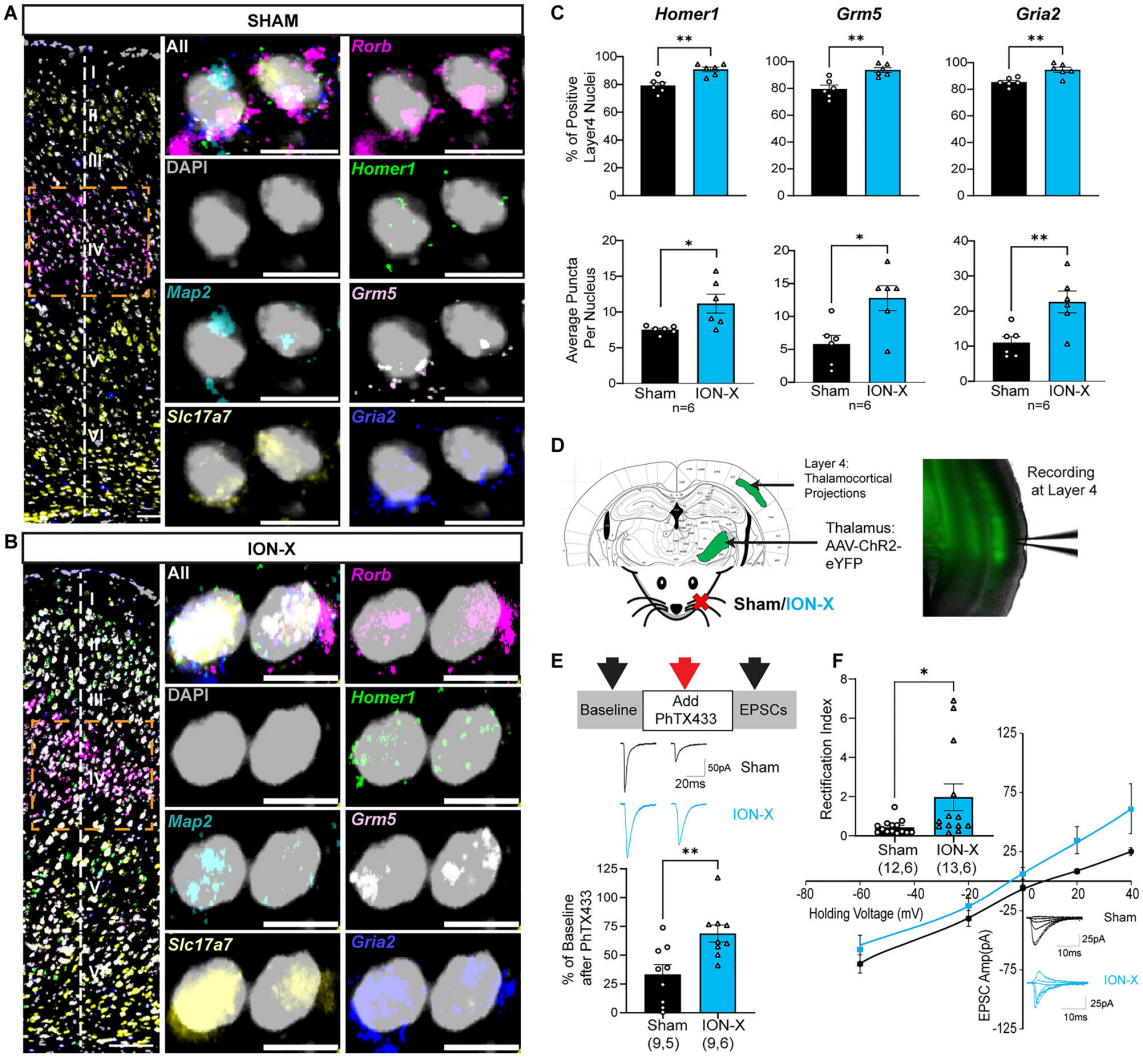
Altered glutamate receptor signaling in the intact sensory pathway after ION-X. ***A***, ***B***, Representative mFISH images showing *Homer1*, *Grm5*, and *Gria2* expression in L4 excitatory neurons from sham and ION-X mice. Scale bars: 100 µm (left panels), 10 µm (right). ***C***, Quantification shows significantly increased expression for *Homer1*, *Grm5*, and *Gria2* in ION-X compared with sham. For each gene, two types of quantification are shown: top graphs show percentage of L4 nuclei positive for that gene, bottom graphs show average number of puncta per nucleus. Each data point represents one mouse (average of two sections/mouse); *n* = 6 mice. Unpaired Student’s *t* test *p < 0.05, **p < 0.01, values are mean ± SEM. ***D***, Schematic showing viral injection of AAV-ChR2-eYFP into thalamus and whole-cell recording from L4 neurons in S1BC to assess GluA2 receptor function at the intact TC synapse. ***E***, TC synapses were less sensitive to PhTX433 after ION-X, Sham *n* = 9 cells, 5 mice, ION-X *n* = 9 cells, 6 mice. Unpaired Student's *t* test **p < 0.01, values are mean ± SEM. ***F***, TC synapses to L4 neurons display increased rectification index after ION-X. Sham *n* = 12 cells, 6 mice, ION-X *n* = 13 cells, 6 mice. Unpaired Student's *t* test *p < 0.05, values are mean ± SEM. See also Figure S2; Dataset S4, and Materials and Methods.

We focused on three genes related to glutamate receptor function that were upregulated in L4 excitatory neurons after ION-X: *Homer1*, *Grm5*, and *Gria2* (adjusted *p* = 1.18 × 10^−15^, 2.76 × 10^−11^, 4.97 × 10^−13^, respectively; Fig. 2*D*, Dataset S3-5). *Homer1* is a scaffolding protein that regulates metabotropic glutamate receptor (mGluR) anchoring and function, including mGluR5 (encoded by *Grm5* mRNA; Tu et al., 1998). *Gria2* encodes for the GluA2 subunit of ionotropic glutamate AMPA receptors (AMPARs), which renders AMPARs calcium impermeable and is important for synaptic plasticity (Rozov et al., 1998; Isaac et al., 2007; Diering and Huganir, 2018). mFISH quantification was performed exclusively in neuronal nuclei, not in other cell types or subcellular compartments such as synapses. Only nuclei that were triple positive for *Map2/Slc17a7/Rorb* (Fig. 3*A*,*B*) were quantified. These mRNA quantification steps were employed to maintain consistency with the snRNA-seq dataset and to ensure inclusion of only verified excitatory neuron nuclei. There was no significant difference between the number of triple positive nuclei in sham or ION-X tissue (Dataset S4-1a). Gene expression quantification was measured as both the percentage of nuclei positive for each mRNA and the average number of mRNA puncta per nucleus (Fig. S2*A–C*). Consistent with snRNA-seq results, genes that were upregulated after ION-X were also increased when measured by mFISH (Fig. 3*C*, Dataset S4-1b). The percent positive nuclei numbers for each gene include the following: *Homer1* (unpaired Student's *t* test: *p* = 0.002, Cohen's *D* = 2.41), *Grm5* (*p* = 0.002, Cohen's *D* = 2.45), *Gria2* (*p* = 0.003, Cohen's *D* = 2.29); puncta per nucleus for *Homer1* (unpaired Student's *t* test: *p* = 0.008, Cohen's *D* = 1.89), *Grm5* (*p* = 0.013, Cohen's *D* = 1.74), *Gria2* (*p* = 0.008, Cohen's *D* = 1.89).

Previous reports have identified heightened plasticity at TC synapses after ION-X (Chung et al., 2017), which may be supported by increased expression of *Gria2* mRNA and subsequent increased expression of functional GluA2-containing AMPARs. To test for the prevalence of GluA2-containing AMPARs at the intact TC synapse, we transfected neurons in the sensory thalamus with an adeno-associated virus expressing channel rhodopsin with a yellow fluorescent protein reporter (AAV9-Syn-ChR2-eYFP) and performed whole cell electrophysiology recordings in L4 neurons two weeks after sham or ION-X (Fig. 3*D*). First, we measured TC sensitivity to Philanthotoxin (PhTX433); a selective antagonist for GluA2-lacking AMPARs. After establishing a stable baseline excitatory postsynaptic current (EPSC) response to LED stimulation of ChR2-expressing TC synapses, PhTX433 was applied to the bath. Twenty minutes after PhTX433 wash-on, the EPSC amplitude in ION-X showed less sensitivity compared with shams (unpaired Student's *t* test *p* = 0.006, Cohen's *D* = 1.48; Fig. 3*E*, Dataset S4-3) indicating that TC to L4 connections contain more GluA2-containing AMPARs after ION-X (Washburn and Dingledine, 1996). To further validate our findings, we tested for the presence of GluA2-containing AMPARs at the TC synapse using a rectification index experiment. The presence of GluA2 renders AMPARs insensitive to polyamine blockade at positive potentials, which is used to measure the rectification index: the ratio of EPSC amplitude evoked at positive versus negative holding potentials (Rozov et al., 1998). At TC to L4 synapses, we found that L4 neurons had a higher rectification index in ION-X compared with shams (unpaired Student's *t* test *p* = 0.044, Cohen's *D* = 0.87; Fig. 3*F*, Dataset S4-3). The presence of GluA2-lacking AMPARs at the TC synapse in sham mice was unexpected, as this type of AMPAR is rare in adult visual TC synaptic connections (Hull et al., 2009; Kloc and Maffei, 2014). In contrast to the visual TC synapse, the somatosensory TC connection in naive adult mice is known to express high levels of GluA2-lacking AMPARs (Goel et al., 2006). Together, our integrated transcriptomic, spatial, and electrophysiological analyses demonstrate that ION-X enhances glutamatergic receptor expression and function at TC synapses in L4 excitatory neurons, including revealing a previously unexplored role for GluA2-containing AMPARs in post-critical period TC plasticity.

### Increased synaptogenesis in intact layer 4 neurons after ION-X

**Figure 4.**
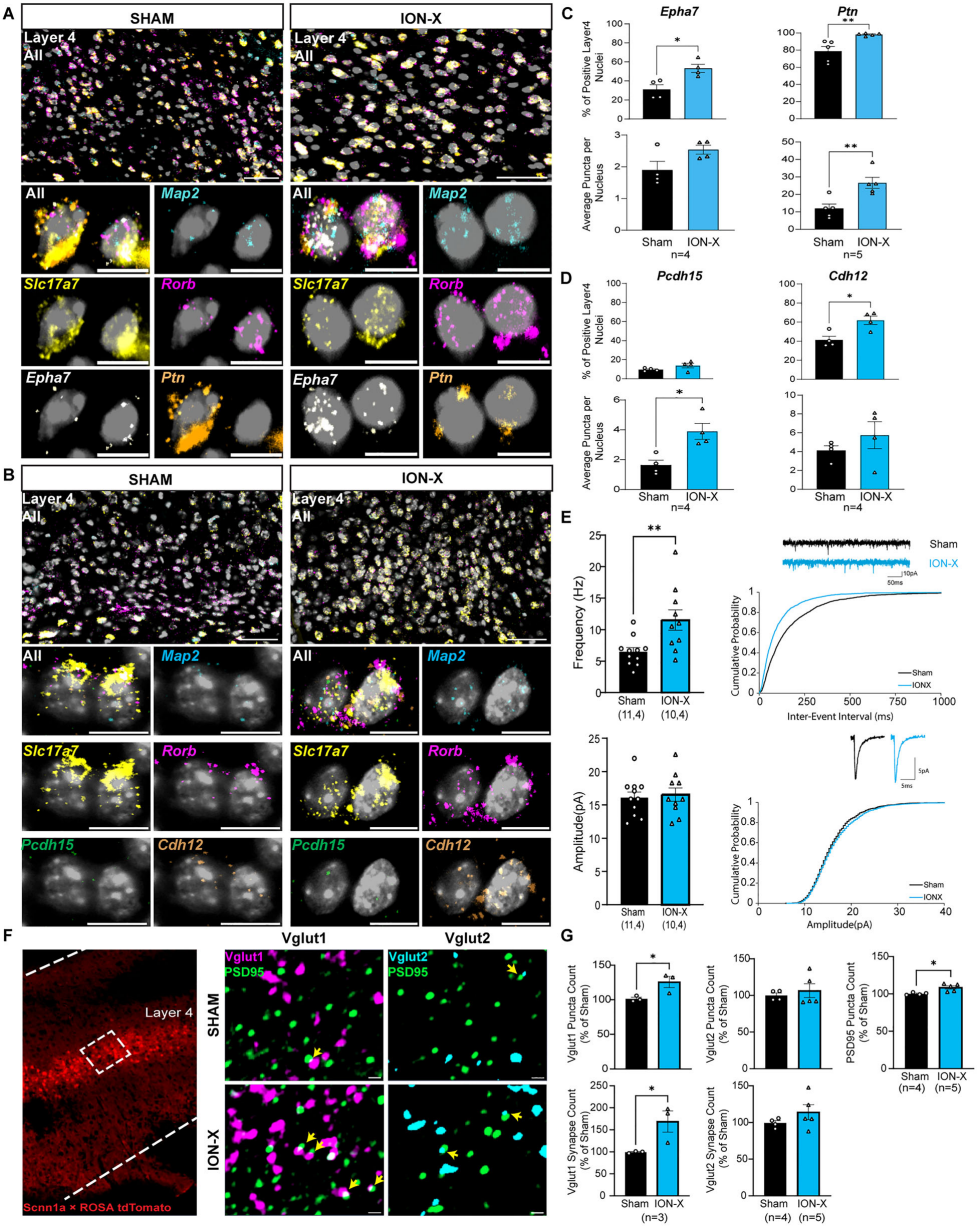
Increased synaptogenesis in intact layer 4 neurons after ION-X. ***A***, ***B***, Representative mFISH images of synaptogenesis related genes: ***A***, *Epha7*, *Ptn*, and ***B***, *Pcdh15*, *Cdh12* in sham (left) and ION-X (right) mice. Scale bars: 50 µm (top), 10 µm (bottom). ***C***, ***D***, Quantification reveals significantly increased levels in ION-X mice. Top, % positive nuclei. Bottom, Average puncta per nucleus. Unpaired Student's *t* test *p < 0.05, **p < 0.01, values are mean ± SEM. ***E***, Miniature excitatory postsynaptic currents (mEPSCs) had increased frequency, but no change in amplitude after ION-X. Sham *n* = 11 cells, 4 mice, ION-X *n* = 10 cells, 4 mice. Unpaired Student's *t* test *p < 0.05, **p < 0.01, values are mean ± SEM. ***F***, Representative IHC images from Scnn1a-tdTomato mice labeling L4 neurons. Synaptic puncta were labeled using Vglut1, Vglut2 (presynaptic), and PSD95 (postsynaptic) markers. Colocalized synapses are indicated by yellow arrowheads. Scale bars: 5 µm. ***G***, Quantification shows significant increase in Vglut1 and PSD95 puncta and their colocalized synapses after ION-X with no change in Vglut2 or Vglut2/PSD95 synapse. *n* = 2–3 mice. Unpaired Student’s *t* test *p < 0.05, values are mean ± SEM. See also Dataset S4.

After unilateral denervation injury, the intact sensory cortex has increased activity in response to intact sensation (Pelled et al., 2009; Yu et al., 2012; Petrus et al., 2020; Sparling et al., 2024). While TC synaptic plasticity may drive the feedforward input, additional connections may further amplify signal propagation within and beyond the cortical column (Jie et al., 2025). L4 excitatory neurons in intact S1BC have significantly upregulated expression of genes related to synaptogenesis (Fig. 2*E*). We focused on four genes that were upregulated in our snRNA-seq dataset: *Epha7*, *Ptn*, *Pcdh15*, and *Cdh12* (adjusted *p* = 1.3 × 10^−4^, *p* = 2.9 × 10^−17^, *p* = 1.4 × 10^−18^, *p* = 0.02, 0.11 respectively; Fig. 2*D*, Dataset S3-5). Ephrins and their receptors support the developmental maturation of synaptic connections (Yun et al., 2003), and *Epha7* plays a critical role in synaptic refinement (Clifford et al., 2014). Pleiotrophin (*Ptn*) regulates neural outgrowth, synapse formation, and synaptic plasticity (González-Castillo et al., 2015; Wang, 2020). *Pcdh15* and *Cdh12* encode for cadherin proteins that promote calcium-dependent cell-cell adhesion (Dalva et al., 2007), which may also enhance activity-dependent synaptic formation. mFISH was performed in intact S1BC, and gene expression was quantified in *Map2/Slc17a7/Rorb* positive nuclei. An overall increase in the expression of these genes was detected in ION-X compared with shams (Fig. 4*A–D*, Dataset S4-1b). The percent positive nuclei for the following genes in ION-X compared to sham include the following: *Epha7* (unpaired Student's *t* test: *p* = 0.014, Cohen's *D* = 2.41), *Ptn* (*p* = 0.009, Cohen's *D* = 2.19), *Pcdh15* (*p* = 0.14, Cohen's *D* = 1.19), *Cdh12* (*p* = 0.01, Cohen's *D* = 2.41). The number of puncta per nucleus for the following genes in ION-X compared to sham include the following: *Epha7* (unpaired Student's *t* test: *p* = 0.08, Cohen's *D* = 1.48), *Ptn* (*p* = 0.007, Cohen's *D* = 2.28), *Pcdh15* (*p* = 0.011, Cohen's *D* = 2.55), *Cdh12* (*p* = 0.33, Cohen's *D* = 0.76).

We identified additional genes and GO pathways related to synaptogenesis in both L4 and L2/3 excitatory neurons. In L4, upregulated genes included *Epha6* and *Epha4* (adjusted *p* = 1.8 × 10^−12^, *p* = 0.001, respectively; Dataset S3-5). Epha4 interacts with multiple ephrin ligands, including Ephrin-B2 (Noberini et al., 2012; encoded by *Efnb2*), which is upregulated in L2/3 excitatory neurons (adjusted *p* = 2.5×10^−7^, Dataset S2-1). Pleiotrophin (*Ptn*) interacts with its receptor, protein tyrosine phosphatase sigma (*ptprs*; González-Castillo et al., 2015), which had increased gene expression in L2/3 excitatory neurons (adjusted *p* = 0.47; Dataset S2-1). Finally, cadherins are known to interact with each other to support synapse formation (Mire et al., 2012; Seong et al., 2015). In addition to *Cdh12*, we identified upregulated *Cdh8*, *Cdh11*, and the synapse adhesion molecule *Cadm1* (Syncam) in L4 neurons (Dataset S3-5). L2/3 excitatory neurons showed coordinated upregulation of five cadherins, five protocadherins, and *Cadm1* (Dataset S2-1). Finally, Robo1, which acts as a brake for TC axon generation (Mire et al., 2012), was upregulated in both L2/3 and L4 excitatory neurons (adjusted *p* = 5.6 × 10^−10^, adjusted *p* = 4.9 × 10^−8^, respectively; Datasets S2-1, S3-5). Together, these transcriptomic patterns suggest that increased synaptogenesis in the intact cortex may arise from intracortical partners, in L2/3 or L4.

To evaluate if the increased synaptogenesis genes yielded more functional synaptic connections, we performed electrophysiological recordings in L4 excitatory neurons. We blocked the known increased activity in intact S1BC (Yu et al., 2012; Chung et al., 2017) by adding tetrodotoxin (TTX) to the recording bath and measured miniature excitatory post synaptic currents (mEPSCs) in L4 neurons 2 weeks after sham or ION-X. mEPSC frequency was significantly increased (unpaired Student's *t* test *p* = 0.014, Cohen's *D* = 1.34), with no change in amplitude or other kinetics (Fig. 4*E*, Dataset S4-3), indicating an increased number of functional synaptic partners after ION-X. To determine the source of these extra synapses, we used Scnn1a-tdTomato mice, which selectively label L4 neurons in S1BC (Fig. 4*F*). Immunostaining was performed for intracortical (Vglut1) or thalamocortical (Vglut2) presynaptic terminals (Fremeau et al., 2001), colocalized with the postsynaptic density marker PSD95 in L4 neurons of intact S1BC two weeks after sham or ION-X (Fig. 4*F*). We detected a significant increase in both Vglut1 and PSD95 puncta and increased colocalized Vglut1/PSD95 putative synapses in ION-X animals compared with shams (Fig. 4*G*). In contrast, Vglut2 puncta and Vglut2/PSD95 colocalized synapses were not different between sham and ION-X mice (Fig. 4*G*). These results suggest that intracortical synaptic connectivity is selectively increased in L4 neurons after ION-X. L4 is known to receive sparse intracortical connections from layer 3 and moderate connections within L4 (Lefort et al., 2009). The upregulation of synaptogenesis genes identifies a previously unexplored mechanism that underlies increased excitatory activity within intact sensory cortex after sensory loss.

### Subtype analysis of layer 4 excitatory neurons

**Figure 5.**
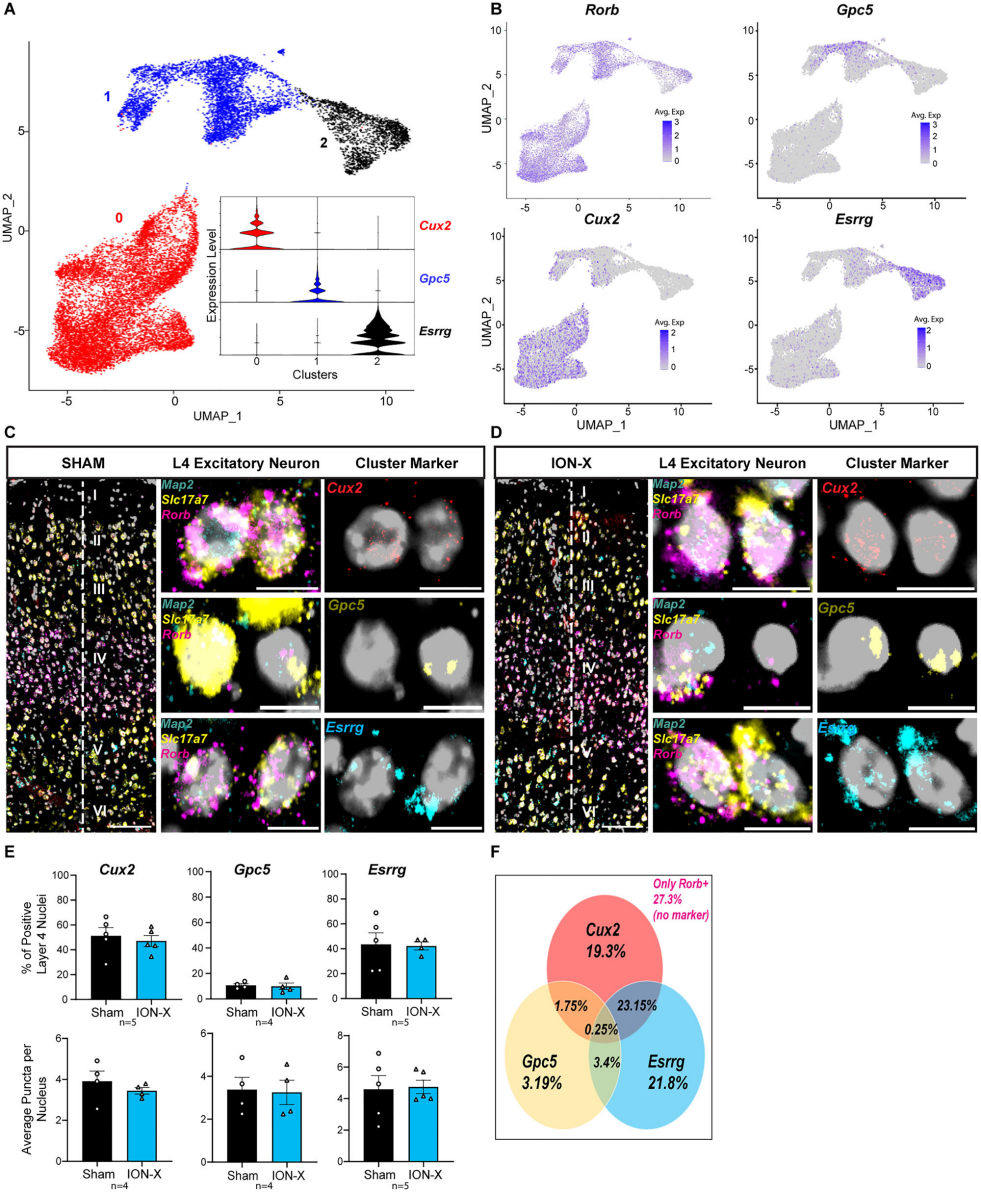
Subtype analysis of layer 4 excitatory neurons. ***A***, Reclustering of L4 excitatory neurons revealed three transcriptionally distinct subclusters, defined by the expression of gene markers: *Cux2*, *Gpc5*, or *Esrrg. **B***, Feature plots with expression of each subcluster marker with L4 marker *Rorb. **C*****, *D***, Representative mFISH images from sham and ION-X mice showing expression of L4 excitatory neurons (*Map2/Slc17a7/Rorb*) and subcluster specific markers: *Cux2* (Cluster 0), *Gpc5* (Cluster 1), *Esrrg* (Cluster 2). Scale bars: 100 µm (left), 10 µm (right). ***E***, Quantification of subcluster marker genes revealed no significant population difference between sham and ION-X. Top, Percentage of nuclei positive expressing each marker. Bottom, Average puncta per nucleus. *n* = 4–5 mice in each group. Values are mean  ±  SEM. ***F***, Venn diagram showing the percentage distribution and overlap among the cluster makers. See also Dataset S3, S4.

We characterized the transcriptional and functional adaptations of L4 neurons in intact S1BC after ION-X. L4 excitatory neurons could be composed of multiple subtypes that exhibit unique adaptations to altered sensory experience. To identify these potential subtypes, we isolated and reclustered the *Map2/Slc17a7/Rorb* positive nuclei from Figure 2*B*. Three transcriptionally distinct subclusters of L4 excitatory neurons were revealed (Fig. 5*A*, Fig. S2*D*), expressing marker genes: *Cux2* (Cluster 0), *Gpc5* (Cluster 1), and *Esrrg* (Cluster 2; Fig. 5*B*). Expression patterns were verified with mFISH (Fig. 5*C*,*D*). The nuclei distribution between subtype clusters was similar between sham and ION-X, suggesting that injury does not alter their prevalence (Fig. 5*E*, Dataset S3-1). Although snRNA-seq data indicated nonoverlapping marker gene expression, mFISH in intact S1BC tissue showed partial coexpression of markers, with ∼28% of nuclei expressing more than one subcluster marker (Fig. 5*F*). These findings highlight the importance of using more than one marker to identify subtypes of nuclei and validating snRNA-seq data with histological methods to accurately determine separate subclusters and their spatial organization within tissue.

### Subtype analysis reveals uniform plasticity across layer 4 excitatory neurons

**Figure 6.**
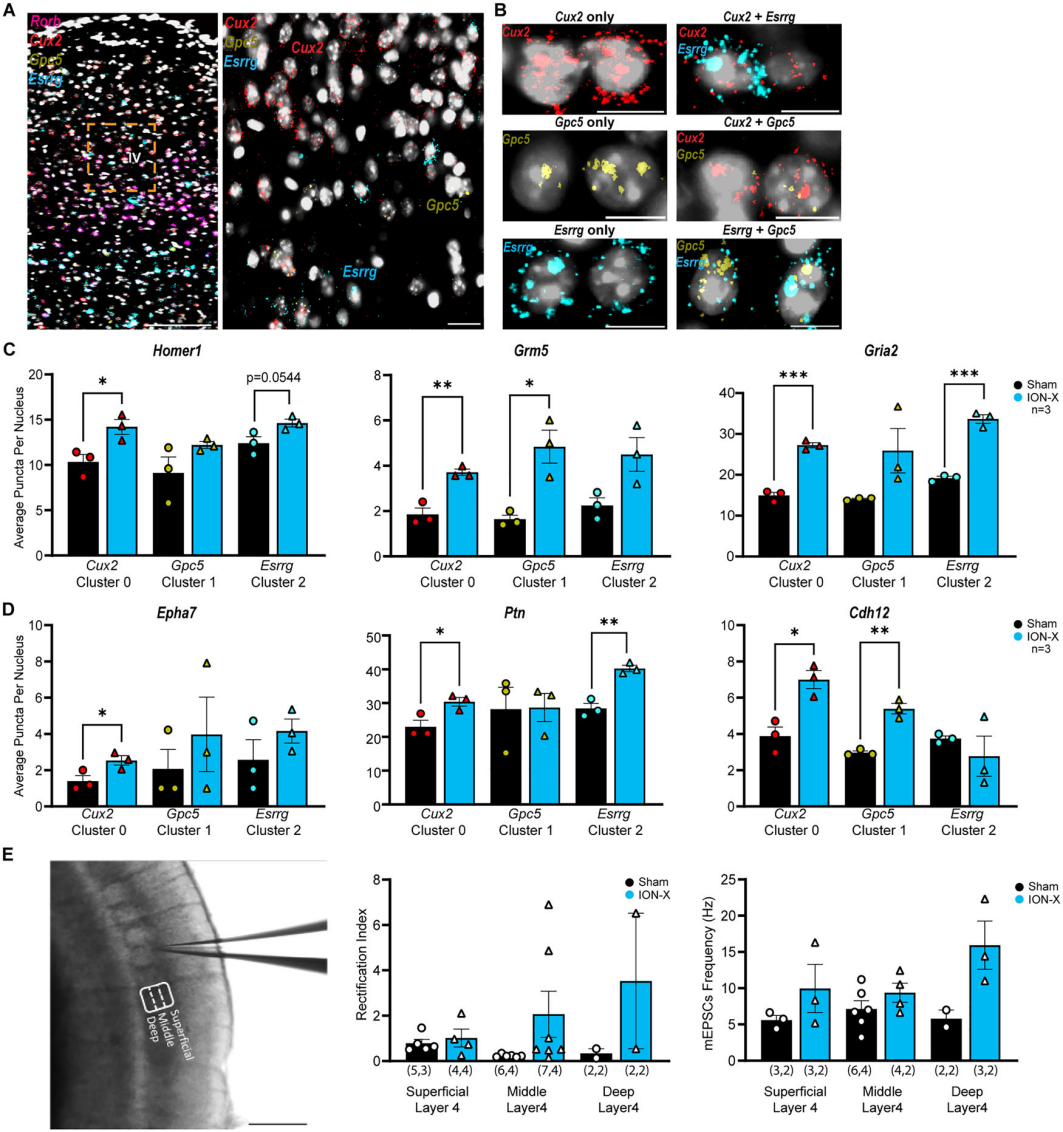
Subtype analysis reveals uniform plasticity across layer 4 excitatory neurons. ***A***, Composite image of S1BC showing coexpression of L4 marker *Rorb* with subcluster markers. The boxed area, expanded on the right, reveals spatial distribution of L4 subclusters: top layer enriched with *Cux2*, bottom with *Esrrg*, and *Gpc5* throughout. Scale bars: 100 µm (left), 20 µm (right). ***B***, Representative mFISH images (scale bars: 10 µm) showing distribution and overlap of cluster markers. ***C***, ***D***, Average mRNA puncta per nucleus for glutamate signaling (***C***) and synaptogenesis genes (***D***) show a homogeneous increase in the gene expression across most subclusters in ION-X. *n* = 3 mice in each group. (unpaired Student's *t* test: *p < 0.05, **p < 0.01, ***p < 0.001), values are mean ± SEM. ***E***, Representative image of patch pipette location in S1BC with depiction of location determination. Barrels are ∼200 µm tall and were divided into three equal segments. Scale bar: 500 µm. Rectification index and mEPSC frequency trends toward increased when the slices are classified according to location in L4. See also Dataset S4.

mFISH detected *Cux2* expression localized superficially in L4, *Esrrg* occupied deeper L4, and *Gpc5* was distributed throughout the layer (Fig. 6*A*,*B*). To assess whether specific subtypes have disparate responses to sensory loss, we quantified the expression of glutamate receptor and synaptogenesis-related genes in nuclei positive for *Map2/Slc17a7/Rorb* plus each nonoverlapping subcluster marker genes *Cux2*, *Gpc5*, or *Esrrg*. Consistent with snRNA-seq data (Dataset S3-2, S3-3, S4), the expression of *Homer1*, *Grm5*, and *Gria2* were generally upregulated in the three subclusters after ION-X (Fig. 6*C*, Fig. S2*E*). Synaptogenesis-related genes (*Epha7*, *Ptn*, and *Cdh12*) also showed increased expression, but the global upregulation after ION-X was less robust than the analysis from all L4 excitatory neurons (Fig. 6*D*, Fig. S2*E*). This finding likely reflects the reduced statistical power due to the smaller number of quantified nuclei in each subcluster versus the whole L4 population. Because subcluster markers were spatially arranged such that Cux2 was superficial and Esrrg was deep L4, we stratified electrophysiology data by recording location. The recording location was determined by visual inspection of images captured during recordings, where the 200 µm deep barrels were divided into three equally spaced 66 µm thick bands: superficial (toward the pia), middle, and deep (toward the white matter; Fig. 6*E*). We found that both GluA2-containing AMPAR function and mEPSC frequency were upregulated after ION-X, though these effects did not reach statistical significance due to the reduced sample size (Fig. 6*E*). These findings suggest a global, nonsubtype-specific, response of L4 excitatory neurons to sensory loss. However, our limited sample size may be missing subtle differences between clusters or cell locations.

## Discussion

Characterizing cell types and their unique roles in sensory circuitry requires the integration of morphology, transcriptional identity, and physiological function analyses. In this study, we used unilateral ION-X in adult mice to trigger post-critical period plasticity and investigate the molecular and functional changes in L4 excitatory neurons of the intact S1BC.

### Adaptations of layer 4 excitatory neurons in sensory cortex

L4 excitatory neurons in sensory cortex differ based on morphology, electrophysiological properties, and gene expression profiles (Codeluppi et al., 2018; Scala et al., 2019; Condylis et al., 2022; Zhang et al., 2023). Their role in sensory processing is important for amplifying feedforward TC inputs (Douglas and Martin, 2004; Yakoubi et al., 2019), and the integration of TC and intracortical inputs in L4 is critical for their unique transcriptional profile during development (Qian et al., 2025). These converging inputs position L4 neurons to play an important role in downstream cortical processing of sensory signaling.

Here we identified three subtypes of L4 excitatory neurons in S1BC. Although these subtypes are transcriptionally distinct, their response to ION-X was largely similar, with DEGs related to glutamatergic signaling and synaptogenesis. These findings indicate that transcriptional heterogeneity and location within barrels do not play a dominant role in how these neurons adapt after ION-X. The complete denervation of sensory inputs in our ION-X model may cause a more homogenous response in L4 excitatory neurons compared with other partial whisker deprivation techniques like checkerboard plucking or spared whisker model (Chen and Brumberg, 2021). ION-X has been shown to increase both synchrony and widespread inhibition of L4 neurons responding to whisker stimulation (Jie et al., 2025). After sensory loss, the role of global circuit activity, neuromodulation, or inhibition may uniformly alter the cortical environment surrounding all three subtypes of L4 excitatory neurons.

### Glutamate receptor signaling

After ION-X, we detected DEGs in L4 excitatory neurons encoding for glutamate receptors and their interacting proteins. Among these, *Gria2*, which encodes the GluA2 subunit of AMPARs, was upregulated. This increased gene expression correlates with increased prevalence of functional GluA2-containing AMPARs at TC synapses. GluA2-containing AMPARs replace GluA2-lacking AMPARs to stabilize recently potentiated synapses (Isaac et al., 2007; Diering and Huganir, 2018), such as those detected in our previous study at the TC synapse after ION-X (Chung et al., 2017). This TC potentiation is thought to underlie increased BOLD fMRI activity observed in the intact cortex after unilateral denervation-induced sensory loss in both rodents (Pelled et al., 2009; Yu et al., 2012; Petrus et al., 2019) and humans (Lotze et al., 2001; Valyear et al., 2020). TC plasticity after sensory loss occurs in three stages: (1) recruitment of silent synapses and TC potentiation (Chung et al., 2017), (2) LTP consolidation with increased GluA2-containing AMPARs (Isaac et al., 2007), and (3) homeostatic adaptations that stabilize neuronal excitability (Whitt et al., 2014; Lee and Kirkwood, 2019). Our snRNA-seq results potentially captured the second two phases.

We detected increased expression for *Grm5* (mGluR5) and *Homer1* (Homer1) in L4 excitatory neurons after ION-X. Perisynaptic mGluR5s (Luján et al., 1996; Scheefhals and MacGillavry, 2018) detect glutamate spillover during high activity and regulate downstream plasticity (Huber et al., 1998). mGluR5s are anchored near the postsynaptic density by Homer 1b/c (Tu et al., 1998); this anchoring is disrupted by the Homer1a isoform during high neuronal activity (Kammermeier and Worley, 2007). Although snRNA-seq and mFISH cannot distinguish between the downstream Homer1 isoforms, the elevated *Homer1* expression observed after ION-X may be dominated by *Homer1a* responding to increased intact S1BC activity (Yu et al., 2012; Chung et al., 2017). The potential roles for Homer1a after ION-X could include neuroprotection against excitotoxicity (Wang et al., 2015) or binding to mGluR5 to enhance NMDA receptor function (Suzuki and Okada, 2010; Marton et al., 2015). Alternatively, Homer1a’s binding to mGluR5 causes constitutive activation of mGluR5 and inhibits NMDAR function (Perroy et al., 2008; Chokshi et al., 2019). This inhibition may homeostatically reduce global (within the neuron) synaptic strength in response to the increased activity (Lee and Kirkwood, 2019; Bockaert et al., 2021). Thus, an increase in mRNA levels for *Homer1* and *Grm5* may both stabilize LTP and preserve neuronal activity within a physiologically normal range. The upregulation of glutamate receptors in L4 neurons likely contributes to synaptic remodeling while homeostatically constraining cortical excitability after sensory loss.

### Functional consequences of intracortical synaptogenesis

SnRNA-seq and mFISH detected upregulated synaptogenesis in L2/3 and L4 excitatory neurons after ION-X. Electrophysiological recordings detected increased mEPSC frequency in L4 neurons, and immunohistochemistry determined that new synapses were preferentially formed at intracortical connections. Combined, these findings indicate that L4 excitatory neurons create new connections after ION-X.

Synaptogenesis has important implications for recovery after injury. For example, after unilateral motor cortex stroke, synaptogenesis in neurons within healthy regions and the unaffected, contralateral cortex correlates with enhanced recovery of the affected limb (Luke et al., 2004; Kraft et al., 2018). Unilateral sensory loss recruits new innervation from neighboring cortical regions (Merzenich et al., 1983; Garraghty and Kaas, 1991; Florence et al., 2000; Yu et al., 2010) which may occur after ION-X. Indeed, this type of synaptic rewiring is thought to enhance recovery by making new connections to overcome stroke (Joy and Carmichael, 2021) or peripheral injuries (Jones and Pons, 1998; Pellicer-Morata et al., 2023).

After ION-X in rats, we have previously reported TC synaptic potency, which hinted toward an increase in the number of TC synapses (Yu et al., 2012). TC synaptic changes are due to a recruitment of juvenile-like plasticity mechanisms, including increased prevalence of silent synapses (Chung et al., 2017). We did not detect increased VGlut2/PSD positive TC synapses, which may be because silent synapses are small filopodia (Vardalaki et al., 2022), beyond the detection abilities of our synapse quantification method. The TC potentiation in mice (Petrus et al., 2019) also correlates with enhanced synchrony in stimulus evoked activity throughout the intact S1BC column (Jie et al., 2025). During sensory circuit development, TC synapses arise, synchronize cortical activity, and trigger the formation of intracortical synapses, which are later refined to effect sparse coding in mature sensory circuits (Molnár et al., 2020; Martini et al., 2021). This sequence of events also appears in the intact S1BC in adult mice after unilateral ION-X. After sensory loss in other modalities, intact TC connections are potentiated and L4 intracortical signaling is concurrently increased, causing a stronger and amplified feedforward sensory signal (Petrus et al., 2014, 2015). This is thought to increase the signal-to-noise ratio to facilitate enhanced perception of intact senses (Petrus et al., 2014). These mechanisms may cooperate to support enhanced perception of intact whiskers to compensate for the loss of the contralateral set.

### Study limitations and avenues of future investigation

This study examined the responses of L4 excitatory neurons to whisker denervation. Although mFISH and electrophysiological data were acquired in male/female balanced samples, the snRNA-seq data set contained only male mice. Because there are known sex-specific transcriptional responses to stress (Walker et al., 2022), plasticity (Gall et al., 2023), and whisker denervation injury (Orczyk et al., 2016, 2017), we likely missed female-specific adaptations to loss of whisker sensation. Future studies must address this knowledge gap.

We identified three transcriptionally distinct subclusters of L4 excitatory neurons. ION-X did not alter the population distribution of these subclusters, nor identify subtype-specific adaptations. The question of how experience influences transcriptional outcomes is of active debate. For example, sensory (visual) deprivation does not alter transcriptional population distributions of visual cortex neurons (Chen et al., 2025), while others have identified increased transcriptional heterogeneity after checkerboard whisker removal (Chevée et al., 2018). Our limited sample size precludes a definitive conclusion, but future studies with increased numbers of molecular markers and complementary spatial transcriptomics could expand upon our findings.

We identified DEGs for glutamate receptor signaling and synaptogenesis in L4 excitatory neurons. Our snRNA-seq dataset and mFISH analysis were restricted to nuclear RNA transcripts and puncta, leaving out important transcriptional adaptations in other neuronal compartments. This is particularly important for mRNAs like *Gria2* (Ho et al., 2014), which are shuttled from the nuclei/soma to axonal or dendritic regions for translation (Horton and Ehlers, 2004; Glock et al., 2021). Future studies may pursue subcellular transcriptional adaptations. Finally, while we have identified new mechanisms by which the sensory cortex overcomes sensory loss, directly testing their causal role will be critical. For example, pharmacological blockade of GluA2-containing AMPARs during the 2 weeks after ION-X could determine if this switch causes the downstream plasticity we observe in both TC strength and synaptogenesis.

### Downstream circuitry and implications for sensory processing

Strengthening responses to intact senses may support recovery after injury. Strengthening the feedforward TC synapses would increase feedforward signal into the cortex, while intracortical connections may further amplify the signal (Jie et al., 2025). Synaptogenesis and glutamate receptor activity likely support these changes, enabling the intact cortex to process remaining sensory inputs more effectively. After unilateral ION-X, mice increase dependence on their intact whisker set to inspect their environment (Wright et al., 2025) similar to humans who rely on their intact limbs after unilateral limb loss (Makin et al., 2013). Our findings reveal critical molecular and synaptic changes that support post-critical period plasticity in adults and provide a basis to harness these mechanisms to support recovery after injury.

## Data Availability

Data generated by single nucleus RNA sequencing is available via the NCBI-based Gene Expression Omnibus and will be available upon publication and request. R-based code for analyses will be available in a publicly available repository that is linked to this publication’s DOI. Any additional information required to reanalyze the data reported in this paper is available from the lead contact upon request. mFISH and electrophysiology data will be shared by the lead contact upon request.
